# Temporal muscle thickness as a preoperative predictor of motor aphasia in Moyamoya disease

**DOI:** 10.3389/fneur.2025.1598387

**Published:** 2025-05-27

**Authors:** Yang Liu, Jie Gao, Gaochao Guo, Yangguang Cui, Lin Zhu, Chaoyue Li, Liming Zhao

**Affiliations:** ^1^Department of Neurosurgery, Henan Provincial People’s Hospital, Zhengzhou University People’s Hospital, Henan University People’s Hospital, Zhengzhou, China; ^2^Public Health Emergency Center, Chinese Center for Disease Control and Prevention, Beijing, China; ^3^Henan Provincial People's Clinical Medical School of Zhengzhou University, Zhengzhou, China; ^4^Department of Pathology, Henan Provincial People’s Hospital, Zhengzhou University People’s Hospital, Henan University People’s Hospital, Zhengzhou, China

**Keywords:** MMD, TMT, MRI, motor aphasia, combination revascularization

## Abstract

**Objective:**

Postoperative motor aphasia is a common complication following left-sided combined revascularization surgery for Moyamoya disease (MMD), yet reliable preoperative predictors remain unavailable. This study evaluates preoperative temporal muscle thickness (TMT), a novel MRI-based parameter, as a predictive biomarker for this complication.

**Methods:**

We retrospectively analyzed 34 adult MMD patients who underwent left-sided combined revascularization between April 2021 and June 2023. Preoperative TMT was measured on axial MRI using multi-planar reformation. Statistical analyses (e.g., *t*-tests) were used to assess the association between preoperative TMT and the incidence, timing, and duration of postoperative motor aphasia.

**Results:**

Excluding complications such as infarction, postoperative aphasia occurred in 28 of 34 patients (82.35%), predominantly pure motor aphasia (25/34, 73.53%), typically emerging on the third postoperative day with a median duration of 4 days. Patients who developed aphasia had significantly greater mean preoperative TMT than those who did not (7.08 ± 1.00 mm vs. 5.70 ± 0.68 mm, respectively; *p* = 0.003). Furthermore, greater preoperative TMT showed a positive correlation with a longer duration of postoperative aphasia (*r* = 0.4907, *p* = 0.0032).

**Conclusion:**

Our findings confirm that TMT independently predicts the occurrence and severity of postoperative motor aphasia in MMD patients after left-sided revascularization. This MRI metric enhances risk stratification, guiding surgical planning and patient counseling. Further studies are needed to validate its utility and explore preventive measures.

## 1 Introduction

MMD is a chronic, progressive cerebrovascular disorder characterized by stenosis and occlusion of the distal internal carotid arteries and the formation of abnormal collateral vascular networks (1). Patients often present with cerebral ischemia, intracranial hemorrhage, cognitive dysfunction, or headaches (2, 3). Combined direct [e.g., STA-MCA (Superficial Temporal Artery to Middle Cerebral Artery) bypass] and indirect (e.g., EDMS) cerebral revascularization is increasingly used to improve cerebral hemodynamics over a wider territory (4–7). However, postoperative neurological complications, including transient or persistent deficits, remain a significant concern following these procedures (8). Specifically, motor aphasia is frequently observed after left-sided combined revascularization, particularly when indirect techniques such as encephalo-myo-synangiosis (EMS) or encephalo-duro-myo-synangiosis (EDMS) are used, involving transposition of the temporalis muscle onto the brain surface (9, 10). While temporal muscle swelling compressing underlying brain structures is a known risk (9, 10), reliable methods to preoperatively identify patients at higher risk for developing postoperative aphasia are currently lacking. This represents a critical unmet need in MMD management as predicting such complications could significantly inform surgical strategy selection and patient counseling.

TMT is readily measurable on standard preoperative MRI scans. While TMT has been explored in other contexts (11), its potential as a specific, non-invasive biomarker to predict postoperative motor aphasia risk following combined revascularization surgery for MMD has not been previously investigated. This study introduces the novel hypothesis that preoperative TMT could serve as such a predictor. We observed that postoperative motor aphasia predominantly occurred following left-sided surgery in our cohort. Therefore, the primary aim of this research was to address the clinical necessity for better risk stratification by determining the predictive value of preoperative TMT for postoperative motor aphasia in MMD patients undergoing left-sided combined revascularization. Establishing this relationship could contribute to more individualized treatment planning and preoperative risk assessment.

## 2 Methods

### 2.1 Patient selection

This study was approved by the institutional review board of our hospital. From April 2021 to June 2023, a total of 34 MMD patients underwent left-sided combined cerebral revascularization (STA-MCA and EDMS) at our institution and were analyzed.

The inclusion criteria were as follows: ① The diagnosis of MMD was confirmed with digital subtraction angiography (DSA) based on the criteria of the Diagnostic Criteria for Moyamoya Disease (12); ② the patient was right-handed and underwent combined revascularization surgery on the left side; ③ the patient had no cognitive impairment and speech disorders before surgery.

The exclusion criteria were as follows: ① pediatric patients (less than 18 years old) and ② patients with postoperative intracerebral hemorrhage, cerebral infarction, and epilepsy.

### 2.2 Measurement of TMT on 3D MRI

**Figure 1:**
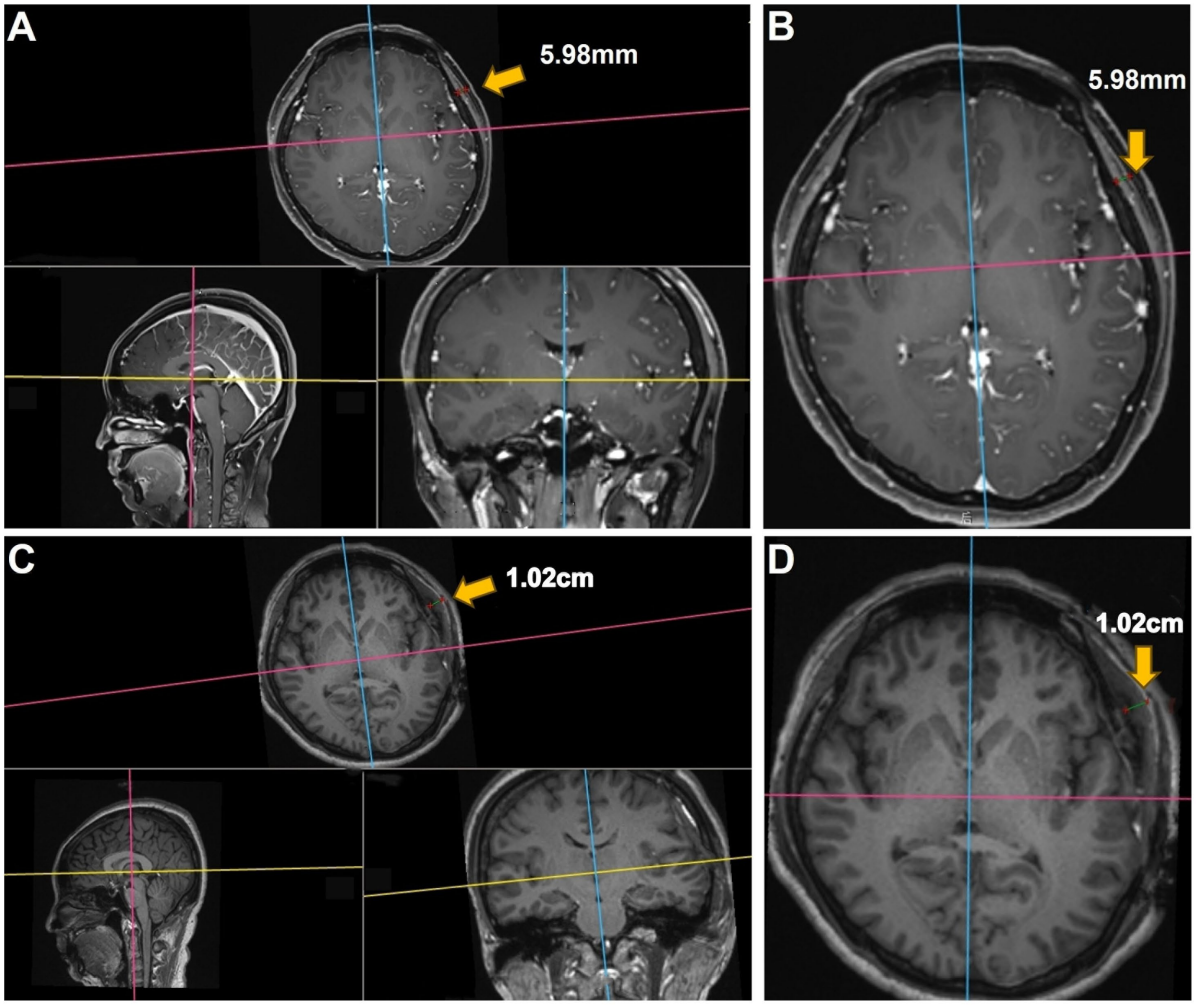
Representative case of motor aphasia due to swollen temporalis muscle. **(A,B)** Preoperative images showing the patient’s left TMT as 5.98 mm. **(C,D)** Postoperative images on day 3 demonstrating swelling of the repositioned temporalis muscle, causing compression of the underlying brain tissue. The postoperative left TMT measured 1.02 cm.

All 3D brain MR images in DICOM were imported into the RadiAnt DICOM Viewer software (Version 4.6.9). The measurement process was performed as described previously (11). We measured TMT of the patients with MMD before and after surgery, as shown in Figure 1.

### 2.3 Operation

**Figure 2:**
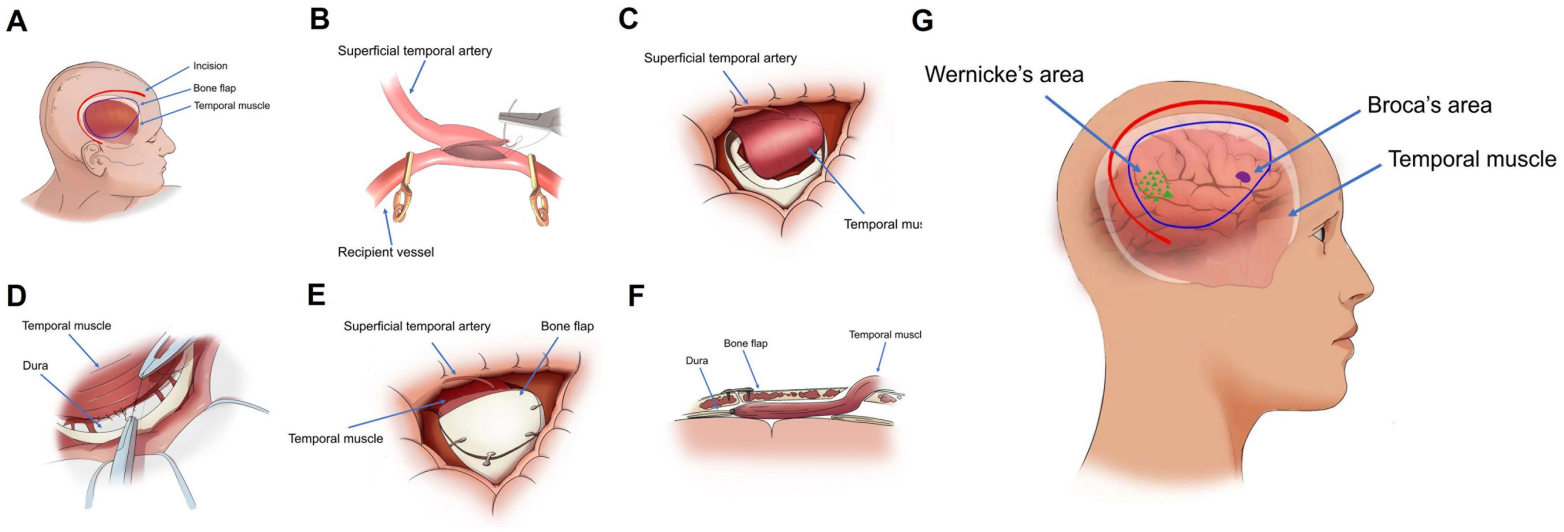
**(A)** Illustration of the surgical site, bone flap localization, and temporal muscle arrangement in the sagittal plane. **(B)** Isolation of the superficial temporal artery, followed by transient occlusion of the superficial middle cerebral artery with an aneurysm clip, prior to end-to-side anastomosis. **(C,D)** Following the bypass procedure, the temporal muscle is attached to the brain surface, and its peripheral edges are sutured to the dura mater. **(E)** Partial removal of the temporal portion of the bone flap, which is then repositioned and secured. **(F)** Postoperative configuration of the bone flap and temporalis muscle in a longitudinal section. **(G)** Schematic representation of the spatial relationships between Wernicke’s area, Broca’s area, and the temporal muscle in a sagittal view.

All MMD patients were treated with combined revascularization, which refers to the simultaneous implementation of STA-MCA and EDMS (Figure 2). STA-MCA is a direct bypass operation that uses a single bypass (the parietal or frontal branch of the STA) as the donor vessel and the M4 segment of the MCA as the recipient vessel for revascularization.

EDMS is an indirect revascularization in which the temporalis muscle is dissected from the bone and then transposed on the surface of the brain to form a new blood supply.

### 2.4 Statistical analysis

Statistical analyses were performed using SPSS Statistics version 25.0 (IBM Corporation, Armonk, NY, United States). Normally distributed continuous variables were expressed as mean ± standard deviation (SD), while non-normally distributed continuous variables were presented as median and interquartile range (IQR). Categorical data were expressed as counts and percentages. For comparisons between two independent groups, the independent samples *t*-test was used for normally distributed continuous data, and the Mann–Whitney U test was used for non-normally distributed continuous data. The chi-square (χ^2^) test or Fisher’s exact test, as appropriate (particularly when expected cell frequencies were low), was used for analyzing categorical data. Spearman rank correlation analysis is used for data that do not follow a normal distribution or for ranked data, to describe the degree and direction of the association between two variables. A *p*-value of < 0.05 was considered statistically significant.

## 3 Results

### 3.1 Baseline information for all patients

The baseline information and characteristics of the patients are shown in Table 1. A total of 34 patients were included in this study, including 18 male (52.94%) and 16 female (47.06%) patients. The average age of the patients was 45.50 ± 10.60 years. The initial symptoms were ischemia in 19 cases (55.88%), hemorrhage in 10 cases (29.41%), and non-specific symptoms in 5 cases (14.70%). The median mRS scores of all patients on admission and at discharge were 0.00 (0.00–1.00). The median mRS scores of patients without postoperative aphasia on admission and at discharge were the same (0.00 (0.00–1.00)). The median mRS scores of patients with postoperative aphasia on admission and at discharge were 0.00 (0.00–1.00) and 0.50 (0.00–1.00), respectively. Fourteen patients (44.12%) had a history of hypertension, 5 patients (14.71%) had a history of diabetes, and 18 (52.94%) had a history of hyperlipidemia. There were no significant differences in age, sex, initial symptom, admission mRS score, past medical history, preoperative Suzuki stage, and discharge mRS score of all patients from matched groups.

**Table 1:** Baseline information statistical table for 34 patients.

Characteristics	Total (*n* = 34)	Language dysfunction		*p*-value
		Yes (*n* = 28)	No (*n* = 6)	
Mean age at operation (years)	45.50 ± 10.60	46.79 ± 10.42	39.50 ± 10.15	0.129
Sex (F/M)	16/18	14/14	2/4	0.660
Clinical presentation				
Ischemic	19 (55.88)	15 (53.57)	4 (66.67)	0.753
Infarction	13 (38.24)	11 (39.28)	2 (33.33)	
TIA	6 (17.65)	4 (14.28)	2 (33.33)	
Hemorrhagic	10 (29.41)	9 (32.14)	1 (16.67)	
Non-specific	5 (14.70)	4 (14.28)	1 (16.67)	
Suzuki stage				
Median	3.00 (3.00 ~ 4.00)	3.00 (3.00 ~ 4.00)	3.50 (2.75 ~ 4.00)	1.000
1	0 (0.00)	0 (0.00)	0 (0.00)	0.257
2	4 (11.76)	3 (10.71)	1 (16.67)	
3	17 (50.00)	15 (53.57)	2 (33.33)	
4	8 (23.53)	5 (17.86)	3 (50.00)	
5	5 (14.70)	5 (17.86)	0 (0.00)	
6	0 (0.00)	0 (0.00)	0 (0.00)	
Admission mRS score				
Median	0.00 (0.00 ~ 1.00)	0.00 (0.00 ~ 1.00)	0.00 (0.00 ~ 1.00)	0.564
0	19 (55.88)	15 (53.57)	4 (66.67)	0.672
1	15 (44.12)	13 (46.43)	2 (33.33)	
Discharge mRS score				
Median	0.00 (0.00 ~ 1.00)	0.50 (0.00 ~ 1.00)	0.00 (0.00 ~ 1.00)	0.365
0	18 (52.94)	14 (50.00)	4 (66.67)	0.840
1	12 (35.29)	10 (35.71)	2 (33.33)	
2	4 (11.76)	4 (14.28)	0 (0.00)	
Past medical history				
Smoking	7 (20.59)	5 (17.86)	2 (33.33)	0.580
Alcohol use	10 (29.41)	8 (28.57)	2 (33.33)	1.000
Hypertension	15 (44.12)	13 (46.42)	2 (33.33)	0.672
Diabetes	5 (14.71)	5 (17.86)	0 (0.00)	0.559
Hyperlipidemia	18 (52.94)	16 (57.14)	2 (33.33)	0.387

### 3.2 Assessment of the incidence of aphasia and its correlation with TMT

**Figure 3:**
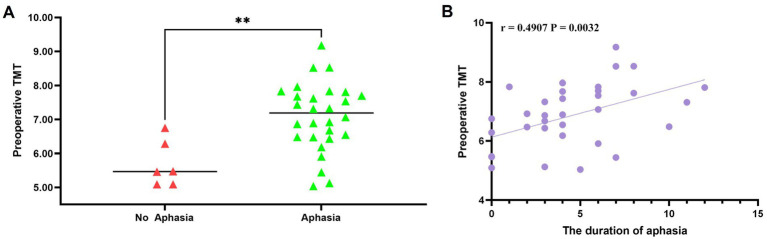
**(A)** Significant association between preoperative left TMT and the occurrence of postoperative aphasia in 34 patients (*p* = 0.003). **(B)** Spearman’s correlation analysis demonstrating a positive correlation between preoperative left TMT and the duration of postoperative aphasia (*r* = 0.4907, *p* = 0.0032).

Aphasia occurred in 28 cases (82.35%) after combined revascularization, of which pure motor aphasia accounted for 73.53% (25 cases) and mixed aphasia accounted for 8.82% (3 cases). The median time of occurrence of aphasia was on the third day, and the median duration of aphasia was 4 days. The mean preoperative left TMT was 6.84 ± 1.08 mm. The median postoperative left TMT was 9.93 (8.71~12.49) mm, and the median change in TMT was 3.27 (2.35~5.42) mm. As shown in Table 2, patients who developed aphasia had significantly thicker temporal muscles before and after surgery than those without aphasia. The mean preoperative left TMT in the aphasia group was 7.08 ± 1.00 mm, significantly greater than 5.70 ± 0.68 mm in the no-aphasia group, with a statistically significant difference (*p* = 0.003) (Figure 3A).

**Table 2:** Analysis of the correlation between TMT and postoperative aphasia.

Characteristics	Total (*n* = 34)	Language dysfunction		*p*-value
		Yes (*n* = 28)	No (*n* = 6)	
Preoperative TMT	6.84 ± 1.08	7.08 ± 1.00	5.70 ± 0.68	0.003
Postoperative TMT	9.93 (8.71 ~ 12.49)	10.02 (9.35 ~ 13.22)	7.72 (7.15 ~ 8.47)	0.000
Changes in TMT before and after operation	3.27 (2.35 ~ 5.42)	4.25 ± 1.89	1.99 ± 0.63	0.007
Lesions were unilateral or bilateral				
Unilateral	3 (8.82)	3 (10.71)	0 (0.00)	1.000
Bilateral	31 (91.18)	25 (89.29)	6 (100.00)	
The right side has been operated on	15 (44.12)	11 (39.28)	4 (66.67)	0.370
Time of occurrence of aphasia	3.00 (2.75 ~ 3.25)	3.00 (3.00 ~ 4.00)	0.00 (0.00 ~ 0.00)	0.000
The duration of aphasia	4.00 (2.00 ~ 6.25)	4.50 (3.00 ~ 7.00)	0.00 (0.00 ~ 0.00)	0.000

In addition, there was a significant difference in the change in TMT between the aphasia group (4.25 ± 1.89 mm) and the non-aphasia group (1.99 ± 0.63 mm) (*p* = 0.007). Spearman’s correlation analysis demonstrated a slight correlation between preoperative left TMT and the median duration of aphasia (*r* = 0.4907, *p* = 0.0032) (Figure 3B).

## 4 Discussion

EDMS is an indirect revascularization technique for MMD utilizing the temporalis muscle and dura as donor tissues. Due to their extensive coverage, EDMS broadly enhances cerebral hemodynamics (13). However, postoperative swelling of the temporalis muscle can compress the underlying brain. This compression sometimes leads to complications such as neurological deterioration, TIA, cerebral infarction, seizures, and aphasia (14).

MMD patients’ brains, already compromised by chronic ischemia and hypoxia, are particularly vulnerable to increased intracranial pressure. Consequently, the mechanical compression exerted by the inserted temporalis muscle may cause focal ischemia in the underlying brain tissue (15). Takemura et al. first described this complication, reporting ischemia beneath the inserted temporalis muscle occurring several days postoperatively (10). Fujimura et al. documented a case requiring revision surgery due to severe muscle swelling (14). They hypothesized that the bone flap wedged the inserted temporalis muscle, trapping its venous vessels and obstructing blood return, which subsequently led to muscle swelling. Separately, Tan et al. identified key brain regions for Chinese language processing, including the middle and inferior frontal gyri (Broca’s area equivalent, motor language) and the inferior parietal lobule, superior temporal gyrus, and temporo-occipital regions (Wernicke’s area equivalent, auditory language) (16).

Interestingly, although the revascularization effect on hemodynamics is maximal immediately post-surgery, aphasia typically does not occur at this early stage. Instead, symptoms often appear 3 to 4 days postoperatively, when many MMD patients undergoing left-sided surgery develop aphasia. This primarily manifests as motor aphasia; sensory language areas are usually unaffected or only minimally involved. Importantly, follow-up MRI scans during this period generally show no evidence of cerebral infarction or hemorrhage to explain these symptoms. While this motor aphasia typically resolves over time, patients may experience restlessness during recovery. Postoperative aphasia is notably rare in children with MMD following combined revascularization. This may be related to their relatively thinner temporalis muscle exerting less pressure on underlying language areas. The delayed onset of symptoms suggests a specific mechanism. Immediately following surgery, the repositioned temporalis muscle does create some initial compression. However, concurrent cerebrospinal fluid (CSF) leakage resulting from dural and arachnoid disruption might initially create space, potentially preventing immediate effects on functional brain areas. Approximately 3 to 4 days post-surgery, this dynamic changes. As CSF dynamics begin to normalize, factors such as inflammatory responses or potential venous congestion (as suggested by Fujimura et al.) (14) can contribute to significant temporalis muscle swelling. This swelling then leads to increased compression on the brain. Broca’s area, being relatively compact and often located closer to the center of the muscle flap, appears more susceptible to this delayed compression than Wernicke’s area, which is typically more anatomically dispersed and distant.

Several strategies aim to mitigate complications from temporalis muscle swelling. Careful surgical dissection that preserves muscle integrity may reduce later swelling. In addition, meticulous shaping of the craniotomy defect (Figure 2E) can minimize skull compression on the muscle, potentially improving venous outflow and decreasing edema. Postoperatively, administering hypertonic agents can lessen muscle swelling and its compressive effect on functional brain areas. Another preventative approach involves sagittal splitting of the temporalis muscle. This technique significantly reduces TMT and may prevent neurological deficits caused by swelling by decreasing muscle volume without compromising the development of collateral vessels (17).

In conclusion, a close relationship exists between TMT and the risk of postoperative aphasia. Preoperative TMT may help predict the potential severity of this complication. Importantly, the risk can potentially be mitigated through modifications in surgical technique and the careful use of postoperative medications.

## Data Availability

The original contributions presented in the study are included in the article/supplementary material, further inquiries can be directed to the corresponding authors.

## References

[1] Ihara M Yamamoto Y Hattori Y Liu W Kobayashi H Ishiyama H. Moyamoya disease: diagnosis and interventions. Lancet Neurol. (2022) 21:747–58. doi: 10.1016/s1474-4422(22)00165-x 35605621

[2] Wang Z Yu J Zhang Y Ruan J Liu X Ma S. A nomogram to predict postoperative new-onset cerebral infarction after revascularization of moyamoya disease in adults and its validation: a retrospective study. Front Neurol. (2025) 16:1537755. doi: 10.3389/fneur.2025.1537755, PMID: 40040915 PMC11876968

[3] Kuroda S Houkin K. Moyamoya disease: current concepts and future perspectives. Lancet Neurol. (2008) 7:1056–66. doi: 10.1016/S1474-4422(08)70240-0, PMID: 18940695

[4] Moussouttas M Rybinnik I. A critical appraisal of bypass surgery in moyamoya disease. Ther Adv Neurol Disord. (2020) 13:1756286420921092. doi: 10.1177/1756286420921092, PMID: 32547641 PMC7273549

[5] Sun J Li ZY Chen C Ling C Li H Wang H. Postoperative neovascularization, cerebral hemodynamics, and clinical prognosis between combined and indirect bypass revascularization procedures in hemorrhagic moyamoya disease. Clin Neurol Neurosurg. (2021) 208:106869. doi: 10.1016/j.clineuro.2021.106869 34419781

[6] Fujimura M Kaneta T Mugikura S Shimizu H Tominaga T. Temporary neurologic deterioration due to cerebral hyperperfusion after superficial temporal artery-middle cerebral artery anastomosis in patients with adult-onset moyamoya disease. Surg Neurol. (2007) 67:273–82. doi: 10.1016/j.surneu.2006.07.017, PMID: 17320638

[7] Wu J Li S Liang R Wang Y Shi F Pan X. Risk factors for perioperative cerebral infarction in moyamoya disease: a meta-analysis. Front Neurol. (2025) 16:1530137. doi: 10.3389/fneur.2025.1530137, PMID: 39926020 PMC11802441

[8] Ohue S Kumon Y Kohno K Watanabe H Iwata S Ohnishi T. Postoperative temporary neurological deficits in adults with moyamoya disease. Surg Neurol. (2008) 69:281–6. doi: 10.1016/j.surneu.2007.01.047, PMID: 17996267

[9] Touho H. Cerebral ischemia due to compression of the brain by ossified and hypertrophied muscle used for encephalomyosynangiosis in childhood moyamoya disease. Surg Neurol. (2009) 72:725–7. doi: 10.1016/j.surneu.2006.10.076 17967485

[10] Noguchi K Aoki T Orito K. Novel Indirect revascularization technique with preservation of temporal muscle function for moyamoya disease Encephalo-Duro-Fascio-Arterio-Pericranial-Synangiosis: a case series and technical note. World Neurosurgery. (2018) 120:168–75. doi: 10.1016/j.wneu.2018.08.171 30196169

[11] Furtner J Berghoff AS Albtoush OM Woitek R Asenbaum U Prayer D. Survival prediction using temporal muscle thickness measurements on cranial magnetic resonance images in patients with newly diagnosed brain metastases. Eur Radiol. (2017) 27:3167–73. doi: 10.1007/s00330-016-4707-6, PMID: 28050694 PMC5491578

[12] Kuroda S Fujimura M Takahashi J Kataoka H Ogasawara K Iwama T. Research committee on Moyamoya disease (spontaneous occlusion of circle of Willis) of the Ministry of Health, labor, and welfare, Japan. Diagnostic criteria for Moyamoya disease – 2021 revised version. Neurol Med Chir (Tokyo). (2022) 62:307–12. doi: 10.2176/jns-nmc.2022-0072, PMID: 35613882 PMC9357455

[13] Imai H Miyawaki S Ono H Nakatomi H Yoshimoto Y Saito N. The importance of encephalo-myo-synangiosis in surgical revascularization strategies for moyamoya disease in children and adults. World Neurosurg. (2015) 83:691–9. doi: 10.1016/j.wneu.2015.01.016 25655688

[14] Fujimura M Kaneta T Shimizu H Tominaga T. Cerebral ischemia owing to compression of the brain by swollen temporal muscle used for encephalo-myo-synangiosis in moyamoya disease. Neurosurg Rev. (2009) 32:245–9. doi: 10.1007/s10143-009-0184-6, PMID: 19159959

[15] Kobayashi K Takeuchi S Tsuchida T Ito J. Encephalo-myo-synangiosis (EMS) in moyamoya disease – with special reference to postoperative angiography. Neurol Med Chir (Tokyo). (1981) 21:1229–38. doi: 10.2176/nmc.21.1229, PMID: 6173785

[16] Tan LH Laird AR Li K Fox PT. Neuroanatomical correlates of phonological processing of Chinese characters and alphabetic words: a meta-analysis. Hum Brain Mapp. (2005) 25:83–91. doi: 10.1002/hbm.20134, PMID: 15846817 PMC6871734

[17] Machida T Higuchi Y Nakano S Izumi M Ishige S Fujikawa A. Sagittal splitting of the temporalis muscle for encephalo-myo-synangiosis to prevent ischemic complications due to a swollen temporalis muscle without inhibiting collateral developments in patients with moyamoya disease. J Neurosurg. (2018) 130:1–8. doi: 10.3171/2018.1.JNS172244 29932376

